# In-Vitro Biofilm Removal Efficacy Using Water Jet in Combination with Cold Plasma Technology on Dental Titanium Implants

**DOI:** 10.3390/ijms24021606

**Published:** 2023-01-13

**Authors:** Rutger Matthes, Lukasz Jablonowski, Lea Miebach, Vinay Pitchika, Birte Holtfreter, Christian Eberhard, Leo Seifert, Torsten Gerling, Rabea Schlüter, Thomas Kocher, Sander Bekeschus

**Affiliations:** 1 Department of Restorative Dentistry, Periodontology, Endodontology, Preventive Dentistry, and Pedodontics, Dental School, Greifswald University Medical Center, Walther-Rathenau-Str. 42A, 17489 Greifswald, Germany; 2 ZIK *plasmatis*, Leibniz Institute for Plasma Science and Technology (INP), Felix-Hausdorff-Str. 2, 17489 Greifswald, Germany; 3 Sirona Dental Systems, Fabrikstraße 31, 64625 Bensheim, Germany; 4 Imaging Center of the Department of Biology, Greifswald University, Friedrich-Ludwig-Jahn-Str. 15, 17489 Greifswald, Germany

**Keywords:** biofilm, cold physical plasma, dental implants, inflammation, PBMC, peri-implantitis

## Abstract

Peri-implantitis-associated inflammation can lead to bone loss and implant failure. Current decontamination measures are ineffective due to the implants’ complex geometry and rough surfaces providing niches for microbial biofilms. A modified water jet system (WaterJet) was combined with cold plasma technology (CAP) to achieve superior antimicrobial efficacy compared to cotton gauze treatment. Seven-day-old multi-species-contaminated titanium discs and implants were investigated as model systems. The efficacy of decontamination on implants was determined by rolling the implants over agar and determining colony-forming units supported by scanning electron microscopy image quantification of implant surface features. The inflammatory consequences of mono and combination treatments were investigated with peripheral blood mononuclear cell surface marker expression and chemokine and cytokine release profiles on titanium discs. In addition, titanium discs were assayed using fluorescence microscopy. Cotton gauze was inferior to WaterJet treatment according to all types of analysis. In combination with the antimicrobial effect of CAP, decontamination was improved accordingly. Mono and CAP-combined treatment on titanium surfaces alone did not unleash inflammation. Simultaneously, chemokine and cytokine release was dramatically reduced in samples that had benefited from additional antimicrobial effects through CAP. The combined treatment with WaterJet and CAP potently removed biofilm and disinfected rough titanium implant surfaces. At the same time, non-favorable rendering of the surface structure or its pro-inflammatory potential through CAP was not observed.

## 1. Introduction

A reliable method to treat peri-implantitis is not available. Microbial residuals impede wound healing and re-osseointegration. Peri-implantitis is an increasing problem for dentists due to an increased number of dental implants in the aging society. Up to 45% of implant patients are affected by peri-implantitis [1]. The inflammation around the implant is accompanied by loss of peri-implant bone. Peri-implantitis is mostly associated with microorganisms embedded in a biofilm on the implant surface and with an imbalance of oral microorganisms and host defence [2], which is considered the main biologic cause of long-term implant failure [3].

The treatment of peri-implantitis is an enormous challenge. While treatment of peri-implant mucositis results in a significant reduction in the degree of peri-implant inflammation in most cases, non-surgical treatment of peri-implantitis often results in a high rate of disease recurrence [4,5]. Surgical treatment then becomes necessary. However, in the oral cavity, access to the implant is hindered. The rough implant surface and the implant threads provide “protected areas” to the biofilm, inaccessible to conventional mechanical removal. For decontamination, different types of mechanical treatment are the preferred method [4,6,7], all with specific shortcomings [4,8,9]. Many implant brands produce a chemically active, micro-rough, hydrophilic surface during the manufacturing process because this modification promotes early healing by cellular interaction in the first phase of wound healing [10,11,12] and leads to improved and faster tissue integration [13]. A major problem with mechanical treatment is that it can destroy these modified implant surfaces [7,14,15] and does not restore hydrophilicity. Even antiseptics as adjuncts to mechanical methods did not yield more satisfactory results [16]. Despite decontamination during flap surgery, about 30% to 40% of cleaned implants experienced further progression of peri-implantitis during a 3-year follow-up [17]. In 7 [18,19,20,21,22,23,24] out of 10 randomised controlled surgical peri-implantitis studies, a gauze pellet soaked with saline or with a disinfectant was used for cleansing in the control group [18,19,20,21,22,23,24,25,26,27]. Thus, at present, cleaning with a gauze pellet seems to be regarded as the gold standard. Yet, a disadvantage of these commonly used therapeutic approaches is that not all bacterial deposits are removed effectively [28,29,30,31]. Compared to ultrasonic instrumentation, superior, non-destructive results were reported for air-polishing with amino acid-based powders [32,33]. However, air polishing, a mix of air, water, and powder, is not approved for surgical interventions because water, air, and powder are applied unsterile, and the air may cause emphysema while performing flap procedures [34,35]. Effective removal of microorganisms or debris from teeth or implants was demonstrated several times, for example, with water pressure [36], high-pressure pulsating water [37], or cavitating systems [38,39] in vitro and with beneficial effects for peri-implantitis treatment in an animal model [40]. The interest in using pressured water for biofilm removal in the dental field is increasing [36,41,42].

To overcome the problem of insufficient instrumentation and the loss of hydrophilicity, an existing high-pressure water jet device [43] was modified (WaterJet), and a cold atmospheric plasma (CAP) device, which will be sequentially applied for surface treatment because of their complementary properties, was developed. CAP has an antimicrobial effectivity [44,45,46,47,48] and can hydrophilize implant surfaces by their reactive compounds, especially reactive oxygen species (ROS), that enhance cell attachment and early healing or osseointegration [13,49,50,51,52,53], also in niches inaccessible for fluids [11,44,54,55]. A meta-analysis on using CAP for bacterial reduction in chronic wounds concluded its safe use without severe adverse events [56,57].

In this study, the WaterJet with and without additional CAP treatment was applied to microbially contaminated titanium implants and compared to treatment with cotton gauze with and without CAP treatment and respective negative and positive controls. Biofilm was generated from subgingival plaque of a single periodontally diseased individual. Biofilm elimination was evaluated by scanning electron micrograph-based quantification and colony-formation units using agar. We hypothesised that adjunctive CAP treatment improves treatment with WaterJet or cotton gauze and that the former outperforms the latter. All treatments were additionally performed on contaminated and sterile titanium discs with peripheral blood mononuclear cells (PBMC from four different donors) to evaluate the inflammatory consequences of the treatments and potential surface modifications on human immune cells known to be involved in tissue inflammation.

**Figure 1:**
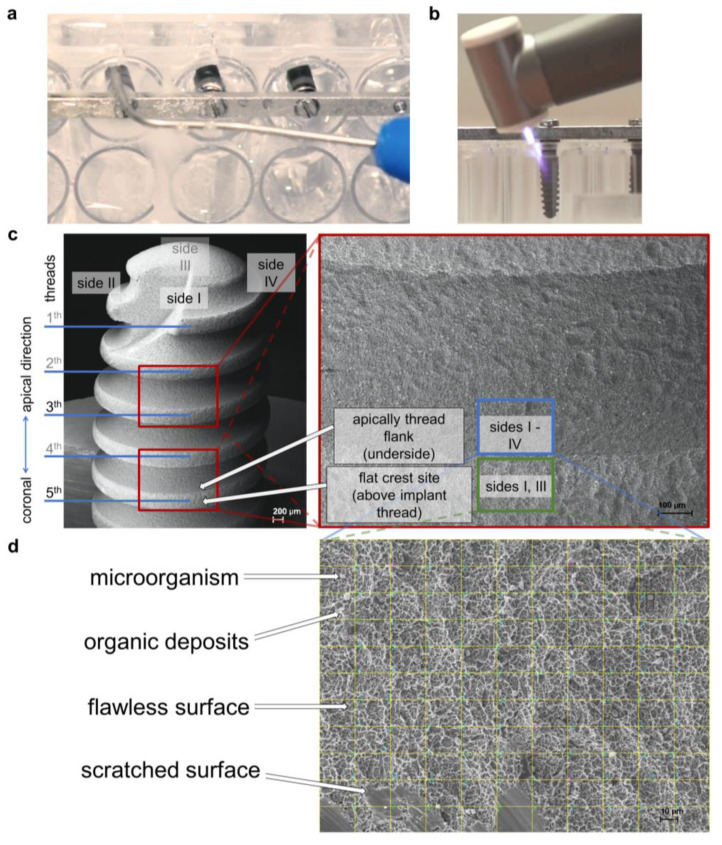
Study setup. (**a**) WaterJet treatment of implant sample in the microtiter plate; (**b**) cold atmospheric pressure plasma treatment of implant sample in the microtiter plate; (**c**) lower magnification scanning electron micrographs showing the implant (scale bar = 200 μm) and the location of threads 3 and 5 (scale bar = 100 μm), where images were taken for later analysis; (**d**) micrograph at 500× magnification with digital grid for quantitative analysis of microbes, organic deposits, flawless implant surface, scratched implant surface, and unknown objects, scale bar = 10 μm.

## 2. Results

### 2.1. Combined Treatment of Dental Water Jet and CAP Provides the Best Decontamination Efficacy

**Figure 2:**
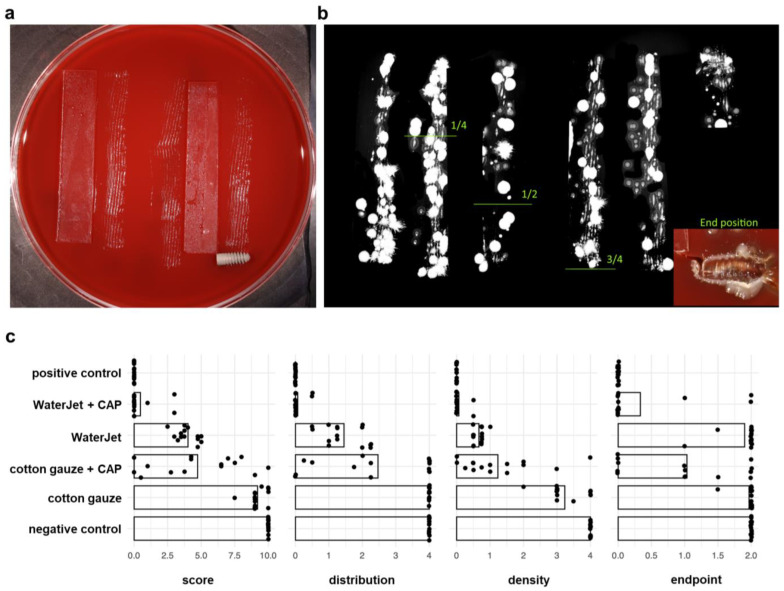
Antimicrobial efficacy. (**a**) scheme of the roll-out-on-agar method, where the implants were rolled on agar along glassware tracks for 30 cm; (**b**) agar plates were incubated for 72 h at 37 °C, and digital images were scored, the gradient limits and the end position are shown; (**c**) score analysis of antimicrobial efficacy.

After rolling the implants on agar plates (Figure 2a), antimicrobial efficacy was assessed by colony-formation analysis (Figure 2b). All score outcomes (distribution, density, final position, and their resulting combined score) revealed the WaterJet + CAP (mean score 0.5) combined treatment to outperform WaterJet (mean score 4.0) alone (88% reduction), cotton gauze alone (mean sum 9.2, 95% reduction), and cotton gauze + CAP (mean score 4.7, 89% reduction). In three endpoint metrics, namely distribution, density, and combined score, WaterJet alone was superior to cotton gauze and cotton gauze + CAP (Figure 2c). The matrix of all data (Table A1) and comparisons between groups (Table A2) underlines these results. The mean score difference between the single and the CAP-combined treatment (3.5 for WaterJet or 4.5 for cotton gauze, reduction of 49%) demonstrated a high but burden-dependent microbicidal effect of CAP. The end position values reflect the cleansing efficacy in the niches of the titanium microstructure. The single score of the end position of the negative control, cotton gauze, and WaterJet was comparable. However, WaterJet showed a lower score than the gauze treatment procedure after subsequent CAP treatment (0.3 vs. 1).

**Figure 3:**
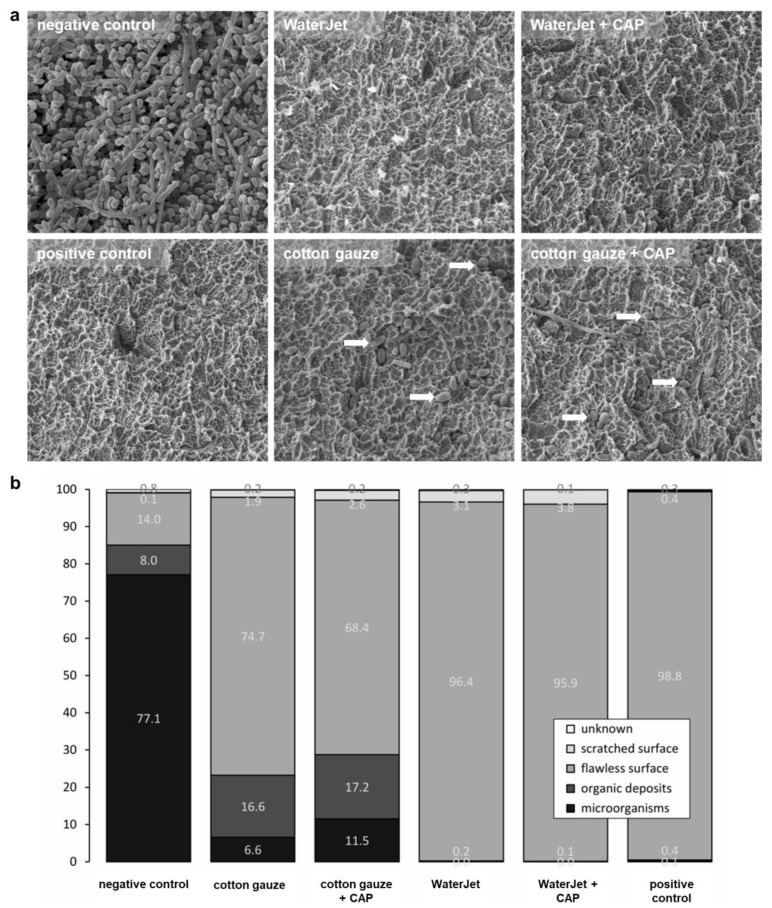
Implant SEM analysis. (**a**) scanning electron micrographs of implant surfaces of untreated samples (negative control), samples treated by WaterJet, cotton gauze, CAP, and sterilized samples (positive control), with white arrows indicating remaining microorganisms; (**b**) percentages of cross points with microorganisms, organic deposits, flawless surface, scratched surface, and unknown among all treatment conditions. Scale bar = 1 µm; white arrowheads show single bacteria.

**Figure A1:**
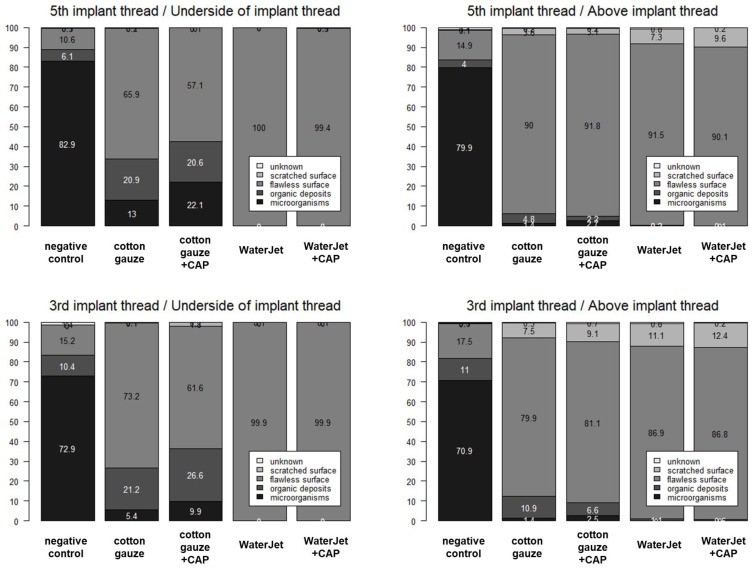
Numbers in mean percentages of microorganisms, organic deposits, flawless surface, scratched surface, and unknown by location (3rd vs. 5th implant thread; underside versus above implant thread) and treatment. CAP, cold atmospheric pressure plasma. Data are presented as median (25% percentile; 75% percentile) and [min–max]. Numbers with difficult readability at the edges of the graphes are mostly between 0–3 and therefore of minor relevance to the overall antimicrobial effect in the respective category indicated in the legends.

**Figure A2:**
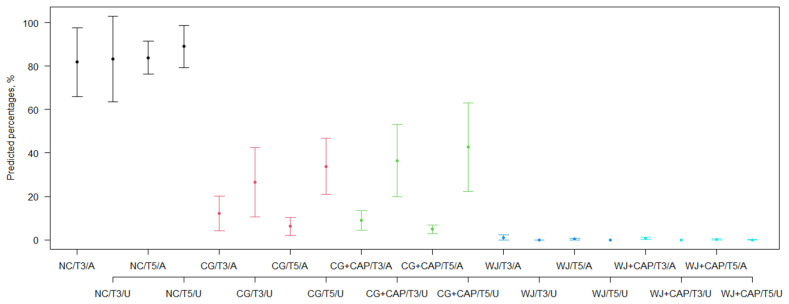
Median of the percentages of ‘microorganisms or organic deposits’ for all combinations of treatment, implant thread (3rd [apical area], T3, vs. 5th [coronal area], T5) and side of implant thread (underside [bottom thread flank], U, versus above [top land of the thread], A) of scanning electron micrographs against the positive control predicted from a generalised binomial regression model with logit link (see Table A2). NC, negative control; CG, cotton gaze; CG + CAP, cotton gauze + cold atmospheric pressure plasma; WJ, water jet; WJ + CAP, water jet + cold atmospheric pressure plasma. Colors refer to different conditions, i.e., control (black), cotton gauze (red), cotton gauze plus plasma (green), waterjet (blue), and waterjet plus plasma (turquoise).

Next, the consequences of the treatments were evaluated by quantifying implant SEM micrographs of the implants’ third and fifth thread features (microorganisms, organic deposits, flawless surface, and scratched surface categorisation), which allow statements about the biofilm removal capacity. Hence, from SEM analysis, a benefit of CAP treatment, as seen with the colony-formation results, cannot be determined because only the biofilm removal but not microbial inactivation is analysable on the scanning electron micrographs. In total, WaterJet and WaterJet + CAP treated surfaces were comparable to the positive control specimens, regarding counts of microorganisms (<0.1%), organic deposits (<0.3%), and surface (>95%) (Figure 3a). Principally similar findings were obtained when focusing on the third and fifth threads of the implants independently of the thread site, but not when focusing both the flat crest site of the thread and the apical thread flank. After gauze with or without CAP treatment, the apically facing site of the thread flank showed a higher number of microbial residues (9 to 16%) and organic deposits (21 to 23.6%) than on the thread’s flat crest site (microorganisms: 1.4 to 2.6%, organic deposits: 4.4 to 7.9%), and in comparison, to water jet treated implants (apically flank facing: <0.1%, crest facing: <0.7%) (Figure A1). The difference in biofilm removal at the apically facing area between water jet and gauze-treated implants is significant (Table A4). No differences were found between the apical (3rd thread) and the coronal implant position (5th thread) regarding all test groups (Table A4). A graphical comparison to show the biofilm removal capacity of the data (here, the features *microorganisms* and *organic residues*, as well as *flawless* and *scratched surface* were combined to *microbial residues* or *implant surface*) based on the statistically predicted values by means of generated data (Figure A2).

Scratched surface area was visible in all mechanically treated implants and ranged between 1.9% in the cotton gauze, and 3.8% in WaterJet +CAP treated implants (Figure 3a). Scratches occurred mainly on the flat crest site (Figure A1). Unknown deposits were found on all samples in comparable numbers independent of the test group. Negative control implants were nearly completely covered with biofilm. In contrast, the positive control implants displayed a pristine surface (Figure 3b). Larger numbers of microbial cells and residues were visible on those contaminated surfaces that were treated with cotton gauze without or with CAP (23.2%/28.7%), while WaterJet without or with CAP treatment showed lower numbers in comparison (0.1%/0.2%). This judgment was independent of additional CAP treatment.

### 2.2. Combined Treatment of Dental WaterJet and CAP Reduced Chemokine and Cytokine Release

**Figure 4:**
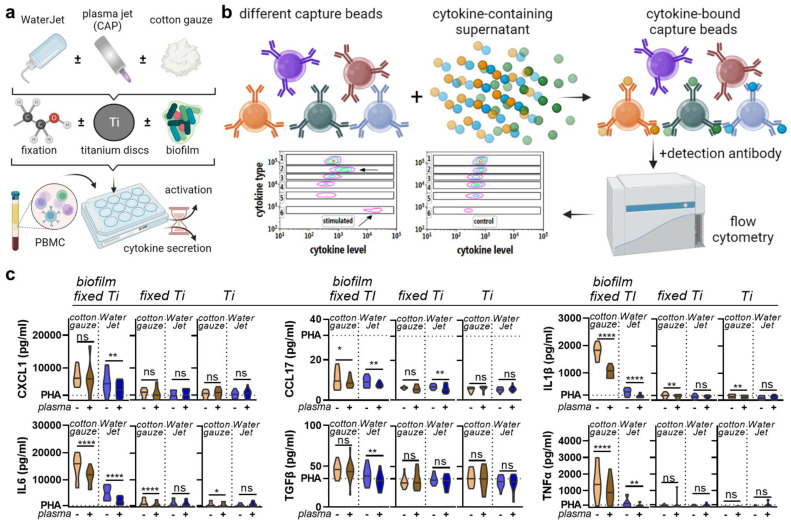
Chemokine and cytokine analysis. (**a**) sampling scheme; (**b**) overview of multiplex assay used in this study; (**c**) absolute analyte concentrations across cotton gauze and WaterJet ±CAP conditions and PHA (dotted line) as reference of leukocyte culture supernatants incubated with contaminated titanium discs (biofilm fixed TI), clean titanium discs exposed to fixative only (fixed Ti), and titanium discs only (no fixation). Principal component analysis across treatments and titanium disc conditions. Data are from four donors and show violin plots and median (**c**). Statistical analysis was performed using paired *t*-test; * = *p* < 0.05, ** = *p* < 0.01, **** = *p* < 0.0001, ns = not significant. PBMC from four different donors were tested. Biofilm was generated from subgingival plaque of a single periodontally diseased individual.

**Figure A3:**
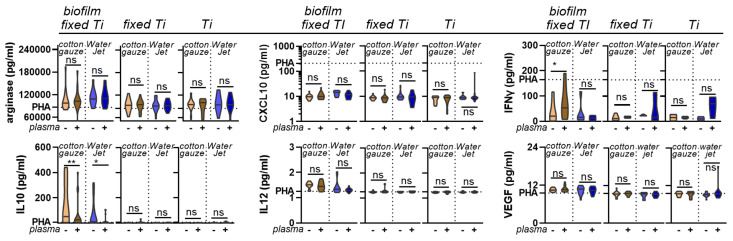
Chemokine and cytokine analysis. Absolute analyte concentrations across cotton gauze and WaterJet ±CAP conditions and PHA (dotted line) as reference of leukocyte culture supernatants incubated with contaminated titanium discs (biofilm fixed TI), clean titanium discs exposed to fixative only (fixed Ti), and titanium discs only (no fixation). Data are from four donors and show violin plots and median. Statistical analysis was performed using paired *t*-test; * = *p* < 0.05, ** = *p* < 0.01, ns = not significant. PBMC from four different donors were tested. Biofilm was generated from subgingival plaque of a single periodontally diseased individual.

The WaterJet + CAP treatment showed superior decontamination efficacy. The next question was whether such treatment might render the biofilm-carrying or sterile titanium surface immunologically active. To this end, human PBMC from four different donors were separately cultured with contaminated (set 1: *biofilm fixed Ti*), non-contaminated but fixed (set 2: *fixed Ti*), and non-contaminated and non-fixed (set 3: *Ti*) titanium discs to infer their inflammatory profiles (Figure 4a) as a measure to assess the safety and tolerability of the CAP treatment by chemokine and cytokine secretion evaluation (Figure 4b). Treatment of biofilm-contaminated discs with WaterJet + CAP significantly reduced CXCL1, CCL7, IL1β, IL6, TGFβ, and TNFα (Figure 4c) as well as IL10 levels (Figure A3). For cotton gauze-treated biofilm-covered discs, CAP added value by significantly decreasing CXCL1, CCL7, IL1β, IL6, IL10, and TNFα levels, while IFNγ was increased. In non-contaminated but cotton gauze or WaterJet-treated titanium discs, the effects of CAP were more modest. If the discs were submerged in fixative before letting them dry and adding PBMC from four different donors separately, CAP exposure led to a diffident but significant decrease of CCL17, IL1β, and IL6. In treated but non-fixed titanium discs, a small but significant reduction in IL1β and IL6 was observed when CAP was combined with cotton gauze, while there were no changes in the case of WaterJet treatment in combination with CAP. Altogether, several analytes were significantly reduced, underlining the profound antimicrobial effects of CAP observed in colony formation assays (Figure 2c).

### 2.3. Combined Treatment with WaterJet and CAP did Not Promote Cellular Inflammation

**Figure 5:**
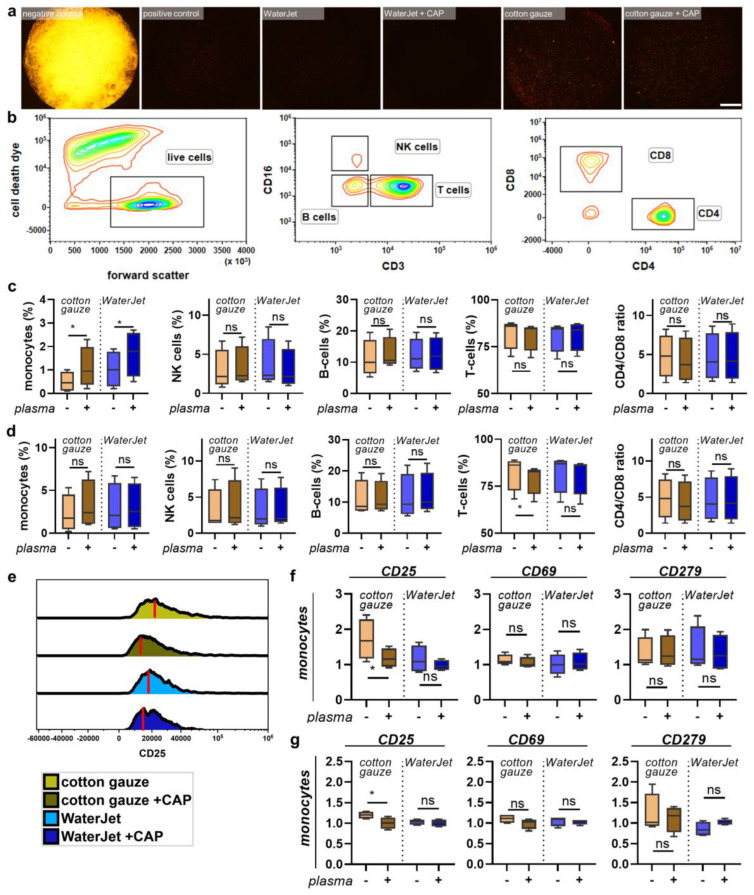
PBMC inflammation marker expression cultured with contaminated and clean, fixed titanium discs. (**a**) representative whole-well fluorescence microscopy images of acridine orange-stained biofilms after various treatments; (**b**) gating strategy showing discrimination of dead cells (left), lymphocyte subpopulations (middle), and T-cell subpopulations (right); (**c**,**d**) percentages of leukocyte subpopulations across all leukocytes cultured on contaminated (**c**) and clean, fixed titanium discs (**d**); (**e**) representative histogram overlays of the surface marker CD25 in monocytes with red lines indicating maximum channels; (**f**,**g**) surface marker expression of monocytes cultured on contaminated (**f**) and clean, fixed titanium discs (**g**). Data are from four donors and show boxplots and median of data normalised to untreated contaminated titanium discs (**f**,**g**). Statistical analysis was performed using paired *t*-test; * = *p* < 0.05, ns = not significant. Scale bar is 1 mm. PBMC from four different donors were tested. Biofilm was generated from subgingival plaque of a single periodontally diseased individual.

**Figure A4:**
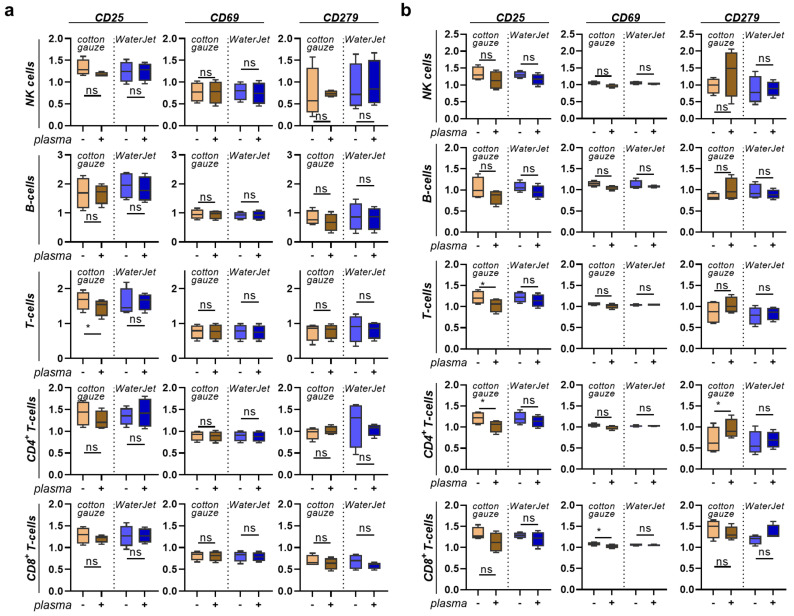
PBMC inflammation marker expression cultured on contaminated or clean, fixed titanium discs. (**a**) percentages of non-myeloid leukocyte subpopulations across all leukocytes in contaminated and cotton gauze, Waterjet, and/or CAP-treated titanium discs with subsequent addition of fixative; (**b**) non-myeloid percentages of leukocyte subpopulations across all leukocytes in clean and cotton gauze, Waterjet, and/or CAP-treated titanium discs with subsequent addition of fixative. Data are from four donors and show boxplots and median of data normalised to untreated fixed titanium discs. Statistical analysis was performed using paired *t*-test; * = *p* < 0.05, ns = not significant. PBMC from four different donors were tested. Biofilm was generated from subgingival plaque of a single periodontally diseased individual.

**Figure A5:**
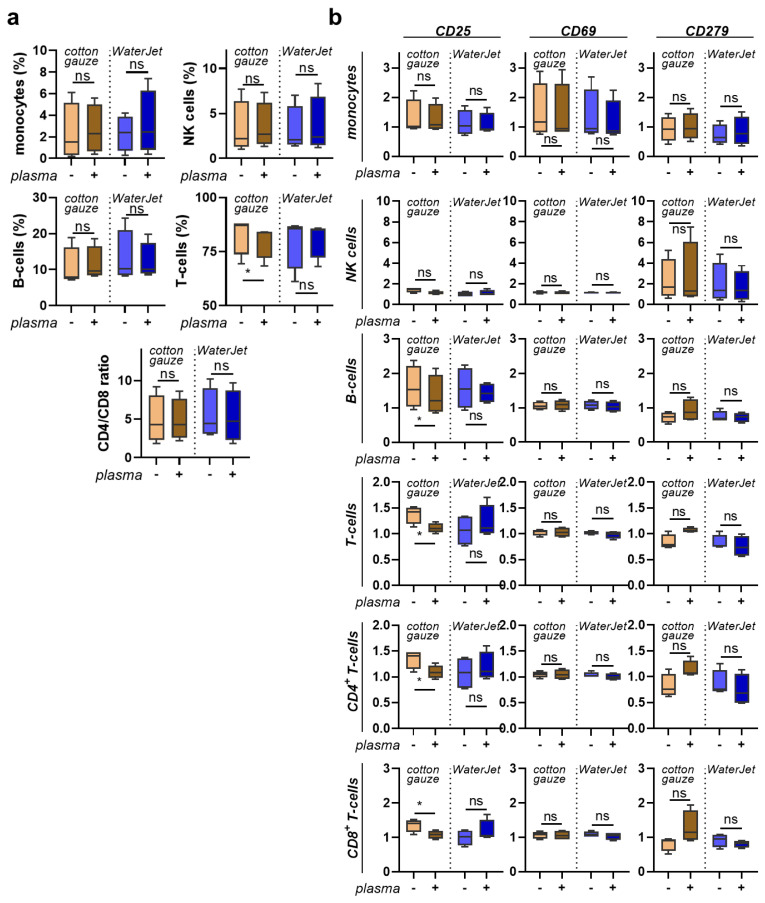
PBMC inflammation marker expression cultured on clean titanium discs. (**a**) percentages of leukocyte subpopulations across all leukocytes in cotton gauze and WaterJet ±CAP-treated titanium discs without subsequent addition of fixative; (**b**) surface marker expression of leukocyte subpopulations and data dependence on titanium disc pre-treatment. Data are from four donors and show boxplots and median of data normalised to untreated titanium discs. Statistical analysis was performed using paired *t*-test; * = *p* < 0.05, ns = not significant. PBMC from four different donors were tested. Biofilm was generated from subgingival plaque of a single periodontally diseased individual.

After mapping the secretion profiles of human leukocytes in contact with the differently treated titanium surfaces, we were interested in analysing the responses of the cells in contact with such surfaces. After all, a novel combination treatment should have not only high efficacy but also good tolerability and should lack immuno-sensitisation. The titanium discs were treated as before, and acridine orange was used to visualise the remaining biofilm (Figure 5a). PBMC from four different donors were cultured on these biofilm discs, which were fixed after treatment to allow non-contaminated co-culture with PBMC, and harvested 24 h later. The cells were stained for several surface markers and gated for several leukocyte subpopulations (Figure 5b). When analysing the percentages of cell populations, cotton gauze + CAP and WaterJet + CAP treatment gave a significant increase of monocytes relative to all leukocytes (Figure 5c). In PBMC cultured with gauze + CAP and WaterJet + CAP-treated and subsequently fixed titanium discs, tendencies were similar for all sub-populations (Figure 5d). Next, all subpopulations were analysed for the late activation marker CD25 (Figure 5e), the early activation marker CD69, and the activation as well as exhaustion marker and immune-checkpoint PD1 (CD279) (Figure A4). Cotton gauze and WaterJet with or without CAP conditions were normalised to biofilm-removal positive control data. CAP + cotton gauze treatment significantly reduced CD25 in monocyte cultured on contaminated (Figure 5e) and clean, fixed titanium discs (Figure 5f). Interestingly, changes in monocyte numbers (Figure A5a) or CD25 (Figure A5b) expression could not be observed in PBMC cultured on cotton gauze and WaterJet with or without CAP treated, unfixed titanium samples. For CD69 and CD279 significant changes could not be observed in any cell type or treatment condition (Figure 5f,g and Figure A5b) except for a small but significant decrease of CD69 in CD8^+^ cytotoxic T lymphocytes cultured on cotton gauze + CAP-treated, unfixed titanium discs (Figure A4b). Regarding the monocyte data on contaminated discs, these data suggested CAP to add to the inherently deficient biofilm removal by cotton gauze, as biofilm-derived microbe-associated molecular patterns active monocytes. In addition, these data indicate that CAP treatment to not induce overshooting inflammatory cellular reactions when combined with WaterJet or cotton gauze for biofilm removal. In sum, titanium CAP treatment does not promote non-specific leukocyte activation marker expression.

## 3. Discussion

Today’s standard treatment for peri-implantitis is the surgical flap procedure, which grants access to implant surfaces to remove microbial biofilms. Such improved access allows better cleaning of the implant threads’ undercuts, as recently shown in an experimental study [68] and long-term patient observations [23]. Hence, surface decontamination is the critical step for inflammation resolution and successful re-osseointegration. Accordingly, the current study aimed to analyse biofilm treatment with CAP combined with WaterJet or cotton gauze to improve peri-implantitis treatment. In addition, we investigated whether such CAP treatment would provoke modifications that lead to non-specific immune cell activation, which would be non-desired in the clinical setting.

Our study identified improved biofilm removal and inactivation efficacy of a novel dental WaterJet in combination with a novel CAP. This test group was compared to cotton gauze treatment, commonly used for peri-implantitis therapy to remove biofilm mechanically and non-abrasively from the implant surface [63]. It was reported as the treatment of choice in several clinical studies, resulting in the successful treatment of implants in about 45% of cases [18,19,23]. The application tip of the Dental water jet handpiece used was designed comparable to periodontal probes with a lateral water outlet to allow perpendicular access to the implant surface, including the apically facing thread flasks. This study complements our former study on the decontamination of titanium discs with promising results for the combined treatment regime with the Dental water jet and CAP in an in vitro setting [58]. For complementation, commercially available implants were used in our experimental set-up with microtiter plates, which hindered free access to the implant surface and thus mimicked the clinical situation in which there is usually limited access to the implant. Importantly, SEM analysis showed that WaterJet cleaning was as efficient as positive controls. Further, the results confirmed that the apically facing sites of implant threads could be sufficiently reached by the WaterJet but not by cotton gauze. This might be the main reason for the underperformance of the latter since gauze treatment’s efficacy is irregular, as previously shown by Ichioka and colleagues on *Streptococcus gordonii*-covered titanium discs [69]. In contrast to our previous study [58], we detected surface alteration on implants treated with the WaterJet application. Probably the constricted access led to direct contact between the nozzle or the tweezer with the gauze pellet and the implant surface, causing scratches. Modifications of the implant microstructure are undesirable because they can reduce corrosion resistance and be cytotoxic to fibroblasts. This was shown before as a less powerful WaterJet showed minor biofilm removal compared to a nylon brush but better-preserved titanium surface structure and corrosion resistance [42].

In our former study [58], we suggested not to analyse the specimen directly after treatment because this approach can overestimate antimicrobial performance, which can be circumvented either by longer incubation [58] or by the cultivation of osteoblasts on treated discs [70]. This approach works with discs but not with implants because it is difficult to protect cleaned from contaminated areas in the latter. In addition, the rough implant surface microstructures act as capillaries and soak fluids, enabling the transport of microorganisms. We could not develop an unfailing method to shield untreated from treated implant areas sufficiently. Therefore, we decided to roll the implant on agar plates where vital microbial cells could attach and proliferate over time. A disadvantage of this method is that microorganisms in deeper niches of the rough structure may not come in contact with the agar or may not be transferred to the agar due to strong adhesion to the implant surface. For the agar roll-out method, we rolled the implants over 30 cm, corresponding to approximately 30 turns of the implants, which is a longer distance than used by Koch et al. [62], allowing to i) raise the chance that microbial cells can attach to the agar, and ii) establish a cells gradient on the agar plates. The density of attached cells in the form of colonies grown, the colony density gradient over the start-to-end distance, and the grown colonies in the final position were the basis for the score established in our study. In some cases, we observed no colony growth on agar, suggesting a very clean or possibly sterile surface. However, colony growth was found in most cases at the end position, where the implant was left on agar, were found in most cases. This underlines the necessity of long-time contact with surfaces or media to detect vital microorganisms in structural niches of micro-rough implant surfaces. Treatment with WaterJet showed a lower score than the gauze treatment procedure, but the score outcome “end position” was comparable, indicating that biofilm removal by the WaterJet is minor regarding the cleansing of structural niches. However, after the subsequent CAP treatment, WaterJet exposure indicates a higher decontamination effect in cavities than after cotton gauze treatment (0.3 vs. 1). The maximum distribution value of 4 (colonies were visible over the total distance of 30 cm) after cotton gauze treatment and the high score (9.2) points to less efficacy than the results of scanning electron images indicate. This could be caused by the more difficult accessibility of the apically facing part of the thread, which dominates the results with the agar-roll method. Nevertheless, WaterJet + CAP could provide a favourable combination treatment for implants of peri-implantitis patients.

The analysis of biofilm removal by scanning electron microscopy images of different positions of the implants was adapted from former studies [33,47,57]. This analysis allowed conclusions about the different cleansing efficacies in different areas of the implant thread, especially its outer surface (crest site) and in the implant threads’ undercuts (apically facing flank site). Improved access to undercuts would be an important improvement compared to currently used methods to remove biofilms during peri-implantitis therapy [7]. Overall, the SEM results confirmed the findings of the colony formation assay.

Additionally, the inflammatory potential after the treatment caused by possible microbial residues was investigated on titanium discs. Until now, in our lab, only the biofilm removal and microbial inactivation were analysed, but not the inflammatory potential of possible microbial residues—an important issue regarding implant decontamination because residues of microbial organic substances act pro-inflammatory [71,72]. In addition, the testing was performed because CAP generates radicals that modify the titanium surface, which could influence the host’s inflammatory response. It is well known that CAP treatment can modulate the immune system [73]. Besides generating ROS in medium and wounds [74,75,76], CAP can modulate inflammatory responses after surface activation by chemical surface modification, for example [77,78]. Our chemokine and cytokine assay results showed the same trend for the majority of analytes assessed. Specifically, mechanical treatment with cotton gauze yielded high inflammatory cytokine secretion, while WaterJet treatment gave lower release levels, consistent with decontamination results by SEM micrograph and colony-forming analyses. Subsequent CAP treatment reduced chemokine and cytokine secretion significantly. In contrast, the discs treated with WaterJet + CAP reached the level of discs without biofilm (referred to as fixed Ti or Ti) for pro-inflammatory cytokines, such as IL1-β, IL-6, and TNF-α. Hence, CAP may not only inactivate microorganisms but also potentially reduces microbial residues that cause the monocyte stimulation to secrete pro-inflammatory cytokines, as recently shown [79]. Regarding the release profiles, it should be kept in mind that this also depends on the type of bacteria in the biofilm [72]. Based on the secretion and surface marker profiles of human leukocytes exposed to treated surfaces without microorganisms involved, we were also able to infer the inflammatory potential of the treatment, which may contribute to the treatment’s safety assessment. Our results suggested that CAP does not promote undesired immune cell activation on treated surfaces under sterile conditions. A consistent decrease of late-activation CD25 in monocytes and mostly lymphocytes was found in several samples in our study, which is in line with a previous report of directly CAP-treated PBMC [80]. While the mechanistic basis of this CD25 decrease is less clear, the amplitude of the effects in our study was small. Functionally, decreased CD25 expression would lead to lower IL2 binding and internalization by leukocytes. CAP-treated surfaces and leukocyte response have been investigated before, albeit to a lesser extent with argon plasma. Oxygen but not nitrogen or air plasma led to a pro-inflammatory response (increased IL1β and TNFα release) [77,81,82]. Additionally, sample fixation did not affect leukocyte response, which is in line with previous findings [83]. A potential limitation is that biofilm was generated from subgingival plaque of a single periodontally diseased individual, which led to a good reproducibility of our results, while it may have also introduced laboratory bias compared to clinical scenarios.

In the literature, air powder devices showed the best cleaning capability of all mechanical methods in an in vitro-setting. However, still, up to 40% of the exposed surface of the implant remains untreated during optimal access, especially in the undercuts of the implant threads [7,84,85]. These areas are inaccessible with air polishing when the spray is directed at 30 to 60° degrees [86]. Our WaterJet has a very thin nozzle shaped like a periodontal probe where a 90° angle of aperture of the broadly fanned (angle approx. 45°) water stream allows the cleansing of the apically facing part of threads, demonstrated by this study.

## 4. Material and Methods

### 4.1. Treatment Setup, Dental Implants, and Titanium Discs

The present study aimed to test whether the combined utilisation of WaterJet with CAP improves biofilm removal from dental implants in vitro. As comparison conditions, mechanical cotton gauze removal alone and combined with CAP was used besides negative (untreated) and positive controls (sterile). For experiments, titanium implants (Ankylos, C/X Implant A9.5 Ø3.5/L9.5; Dentsply Sirona Implants, Mannheim, Germany) were used. The implants were fixed in a special holder and were autoclaved (20 min at 102 °C) before biofilm cultivation. The implants were fixed in line on bars (Figure 1a–c) designed for placing the samples in a 48-well microplate (precision mechanics of the workshop of the Greifswald University Medical Center, Germany). The bars were placed in a 48-well microplate (Techno Plastic Products, Trasadingen, Switzerland) for biofilm cultivation and implant treatment procedures. The cavity of the wells with a diameter of 10.6 mm hampered access to implant surface treatment. This mimicked intraoral therapy and contrasted freely accessible surface treatments often used in laboratory investigations that were not reflecting the clinical situation. For experiments on the inflammation potential of biofilm residues treatment procedures with CAP, sand-blasted, acid-etched sterile titanium discs (DOT, Rostock, Germany) with a diameter of 5 mm, a thickness of 1 mm, Ra = 1.23 µm, and Rq = 1.53 µm (measured using a Dektak^3^ST Surface Profilometer; Veeco, Irvine, CA, USA) were used.

### 4.2. Biofilms Cultivation

Subgingival plaque was collected with curettes from deep pockets of the same periodontally diseased volunteer for each of the three experimental runs. Using biofilm from only a single individual led to greater reproducibility of our results, while it may also introduce laboratory bias compared to clinical scenarios. It was placed in a tube with the culture media Schaedler Broth (Carl Roth, Karlsruhe, Germany) and incubated for 24 h at 37 °C to serve as inoculum for biofilm cultivation. Plaque removal and collection for study purposes was approved by the ethics committee of the University Medicine Greifswald (ethical approval registration number: BB 094/19). The biofilm cultivation on implants was set up in the above-mentioned microtiter plate model (Figure 1a), covered with initially 1250 µL pre-incubated plaque suspension, and cultivated for 7 days on a shaker (Titramax 1000 with incubator 1000; Heidolph, Schwabach, Germany) at 37 °C. The medium was replaced every 24 h, whereas the volume was decreased by 100 µL each of the first five times. For the sixth time, the medium was replaced again with 1250 µL. After cultivation, the medium was removed, and the biofilm-covered implants were transferred into a new microtiter plate for the experiment. The biofilm cultivation on titanium discs took place in 96-well microplates (Techno Plastic Products, Trasidingen, Switzerland). The discs were placed into wells, covered with 100 µL pre-incubated subgingival human plaque suspension, and cultivated in broth for 7 days at 37 °C and 5% CO_2_. The medium was replaced every 24 h. After cultivation, the medium was removed.

### 4.3. Physico-Chemical Treatment with Cold Atmospheric Pressure Plasma (CAP)

CAP treatments were performed under an exhaust hood. The plasma jet (periINPlas, developed by the Leibniz Institute for Plasma Science and Technology (INP), Greifswald, Germany) was operated at a frequency of 0.95 MHz at 2-3 kV_pp_ and 1.6 W maximal input DC-power [58]. The noble gas argon (ALPHAGAZ 99.999% purity; Air Liquide, Düsseldorf, Germany) was used as carrier gas at a flow rate of 2.3 standard liters per minute precisely controlled by a mass flow controller (MKS Instruments, Munich, Germany). The plasma jet has been developed as a medical device, i.e., a risk management file exists, and safety tests have been carried out under the appropriate ISO and IEC standards [58]. The CAP handpiece was designed to fit into a dental handpiece. The CAP discharge properties were comparable to the formerly used plasma source kINPen 09 [32]. The ability of CAP to enhance the wettability on titanium implant surfaces was previously presented [58], which supports human cell attachment and possibly subsequent healing processes [46,52,59,60,61]. For implant treatment, the CAP device was hand-held and moved in angles between 20° and 90° horizontally and vertically along the implant (fixed in the microtiter plate model, see above) for 120 s (Figure 1c). Implants were stored temporarily in isotonic saline solution until further experimental steps or stored after fixation in 2.5% glutaraldehyde in PBS at 4 °C. For titanium disc treatment, a computer-controlled x/y/z stage (micos; SMC Corvus eco, Irvine, CA, USA) directed the specimen holder in meandering movement at a distance of 5 mm under the handpiece nozzle [58]. The specimen surface was scanned 5× with a speed of 2 mm/s corresponding to 58 s treatment time for the total surface of one disc side (20 mm^2^). The disc edge was CAP-treated during the meandering motion through the broad plasma effluent. Both disc sides were treated.

### 4.4. Analysis of Implant Biofilm Removal by Scanning Electron Microscopy

For scanning electron microscopy (SEM), the fixed implant samples (see above) were washed three times with PBS for 5 min each, two times in deionised water for 5 min each, and then dehydrated in a graded series of aqueous ethanol solutions (10%, 30%, 50%, 70%, 90%) and 100% on ice for 15 min each step. The samples were then allowed to reach room temperature before the ethanol was replaced with a new 100% ethanol solution at room temperature for 10 min. Subsequently, implants were critical-point-dried with liquid CO_2_. Finally, implants were fixed in a pin specimen holder with a slot and retaining screw (Plano, Wetzlar, Germany), sputtered with gold/palladium, and examined with a scanning electron microscope EVO LS10 (Zeiss, Oberkochen, Germany). All micrographs were edited using Adobe Photoshop CS6. Of each implant, 12 micrographs (magnification 500×) were captured. Here, two micrographs from each of the 4 implant sides, respectively rotated by 90°, were taken each at the apically facing sites of threads 3 and 5. Two additional micrographs were taken from flat crest sites of threads 3 and 5 of implant sides 1 and 3, respectively (Figure 1d). Data were randomised and blinded before analyses by two individuals independently using ImageJ (v1.50; US National Institutes of Health, Bethesda, MD, USA). Each treatment modality (four test groups and two controls) was performed with a total of 9 implants with 3 runs. To avoid bias of surface analysis, a rectangular grid was overlaid on the images with 10 × 10 crossing points (ImageJ plugin “Grid Overlay”). At each crossing point, the surface characteristic was evaluated to determine whether a microbial cell, blank implant surface, scratched implant surface, organic deposits, or unknown deposits (Figure 1e) were present (plugin “Cell Counter”), resulting in 800 spots per implant for the apical thread facing sites, and 400 spots for the flat crest sites of the threat, resulting in a total number of 10,800 spots per test group. Results are given as the percentage of crossing points, corresponding to the total number of crossing points because 100 crossing points per image were counted. Microorganisms and organic deposits data were merged for statistical biofilm removal efficacy analysis, as both are equally relevant.

### 4.5. Analysis of Implant Decontamination by the Roll-Out-on-Agar-Method

Implants were placed on Columbia Agar with 5% sheep blood (Becton Dickinson, Heidelberg, Germany). With sterile medical gloves (Semperit, Vienna, Austria), the implant was rolled for 30 cm on the agar along glassware tracks and two agar plates per sample. Considering the implant radius and the entire rolling distance, the procedure correlated to 30 implant turns (Figure 2a). The implant was left at its end position for incubation. The agar plates were incubated for 72 h at 37 °C. This technique was adapted from Koch et al. [62]. For data analyses, images of each agar plate were acquired using a digital camera (Canon EOS 450D, Macro lens EF-S 60 mm 1:2.8; Canon, Krefeld, Germany). The images show the distribution and density of grown colonies along the roll-out line (Figure 2b). To evaluate the data, an evaluation system was defined containing three single scores: (i) distribution of colonies and (ii) density of grown colonies along the roll-out line, and iii) growth of colonies at the final position where the implant was left, with categories ranging from value 0 to 4 (i and ii) or from 0 to 2 (iii). The categories are (i) no colonies from start to end (0), sporadic colonies and no distribution gradient (0.5), colonies only in the first quarter (1), colonies in the first and second quarter (2), colonies until the third quarter (3), colonies in all quarters along the roll-out line (4); (ii) no colonies (0), sparsely sporadic colonies (0.5), sporadic colonies (1), predominantly single colonies (2), dense colonies with sparsely single colonies (3), dense colony layer (4); (iii) no colonies (0), 1 to 3 colonies (1), dense colony cluster (2). The score outcomes were determined by two individuals independently. Every single score and the sum of the three scores (combined score) was used for statistical analysis. Each treatment modality (four test groups and two controls) was performed with a total of 15 implants with 4 runs.

### 4.6. Inflammatory Profiling

Titanium discs, placed in 96-well plates, were either left original and sterile or inoculated with biofilm. After cotton gauze, WaterJet, and/or CAP treatment of the titanium discs, the latter were incubated in ISS for 2 h. Afterward, the contaminated discs and one set of sterile discs were fixed with a formaldehyde solution (4.5%) for 24 h. The third set of sterile titanium discs was not fixed. Then, all three sets were washed with and stored in 50 µL of ISS. For experiments, to each well, 50 µL fully-supplemented Roswell Park Memorial Institute (RPMI) 1640 cell culture medium (Pan-Biotech, Aidenbach, Germany) containing 10% fetal bovine serum (Sigma-Aldrich, Taufkirchen, Germany), 1% glutamine (Corning, Kaiserslautern, Germany), and 1% penicillin/streptomycin (Corning) were added harbouring 1 × 10^5^ peripheral blood mononuclear cells (PBMC). The cells were isolated as previously described [63]. Four different donors were tested. To some samples, 100 ng/mL phytohemagglutinin (PHA; Sigma-Aldrich) was added as a positive control. PBMC were cultured for 24 h with titanium discs of set one (contaminated but exposed to fixative after the treatments to allow subsequent co-culture with immune cells), set two (sterile but exposed to fixative), and set three (sterile and not exposed to fixative). In the first set, the idea was to test the impact of CAP treatment on the biofilm and the subsequent inflammatory consequences. In the second set, the question was whether CAP treatment led to surface alterations on the titanium discs, which induced possibly unintended inflammatory reactions in immune cells. To compare this experiment with set 1, a fixative was added after treating the titanium discs. To infer the consequences of CAP treatment of titanium discs on PBMC without the addition of fixative (which could have possibly altered potential residues introduced by CAP), set 3 was set up and tested. PBMC from four different donors were tested. Discs of all sets were left untreated (controls) or exposed to cotton gauze, cotton gauze + CAP, WaterJet, or WaterJet + CAP. Subsequently, each well’s solution was mixed, and cells were harvested and centrifuged. Supernatants were collected and stored at −20 °C for later analysis. Cell pellets were washed in PBS and stained with monoclonal antibodies conjugated to fluorochromes targeting CD3 (BV510), CD4 (APC/Cy7), CD8a (AF700), CD11c (PE/Cy7), CD14 (BV650), CD16 (PerCP/Cy5.5), CD25 (PE), CD69 (BV421), and CD279 (APC) (all BioLegend, Amsterdam, The Netherlands). For dead cell inclusion, iFluor 860 maleimide (Biomol, Hamburg, Germany) was added. After 15 min of incubation at room temperature in the dark, cells were washed and resuspended in FACS buffer. Cells were acquired using a CytoFLEX LX flow cytometer (Beckman-Coulter, Krefeld, Germany) equipped with 365 nm, 405 nm, 488 nm, 561 nm, 633 nm, and 808 nm laser diodes. Data analysis was performed using Kaluza acquisition 2.1.3. software (Beckman-Coulter). For parallel analysis and quantification of several cytokines and chemokines in cell culture supernatants, the LEGENDplex (BioLegend) multiplex bead technology was performed as described before [64]. In short, supernatants were mixed with capture beads and antibodies, washed, and beads were acquired utilising a CytoFLEX S (Beckman-Coulter) flow cytometer that was equipped with a 96-well plate autosampler. Total cytokine and chemokine concentrations were calculated against a 5-log curve fitting calculated from a 7-fold serial dilution series using LEGENDplex software (BioLegend) for the analytes arginase, C-X-C motif chemokine (CXCL) 1 (GRO1/GROα), CXCL10 (IP-10), CC chemokine ligand (CCL) 17 (TARC), interferon (IFN) γ, interleukin (IL) 1β, IL6, IL10, IL10, tumor growth factor (TGF) β, tumor necrosis factor (TNF) α, and vascular endothelial growth factor (VEGF). Statistical analysis was done for all three titanium disc sets between cotton gauze vs. cotton gauze + CAP and WaterJet vs. WaterJet + CAP for 3 discs per donor resulting in 12 samples of each test group and disc sets.

### 4.7. Statistical Analyses

The single implant or disc was the statistical unit. Descriptive SEM data and agar analysis results of treated implants were presented as medians with 25% and 75% quantiles and minimum and maximum values. For SEM, generalised binomial regression models with logit link were used to estimate the effects of ‘treatment’ (reference: negative control), ‘implant thread’, and ‘site of implant thread’ on the percentage of cross points showing ‘microorganisms or organic deposits’ versus ‘flawless or scratched surface or unknown’. The model included two- and three-fold interaction terms and all main terms. Beta-coefficients with 95% confidence intervals (CI), corresponding odds ratios with 95% CIs, and *p*-values were reported. Predicted percentages (with 95% CIs) of ‘microorganisms and organic deposits’ were plotted. *p*-values < 0.05 were considered statistically significant. Statistical analyses were conducted using Stata/SE 17.1 [65] and R 4.2.1 [66]. For the agar results, firstly, pairwise comparisons were performed using Mann-Whitney-U Tests. Secondly, simple linear regression models were constructed to estimate the effects of different treatment methods (negative control or biofilm as the reference) on all three score outcomes (colony distribution, colony density, colony growth at the position, and the combined score) adjusting for samples and test runs and the Beta-coefficients, their corresponding 95% CIs, and *p*-values were calculated. Finally, post hoc comparisons between groups using linear combinations of regression coefficients were performed. *p* values < 0.05 were considered statistically significant. Analyses were conducted using Stata/SE 14.2 (StataCorp., College Station, TX, USA). Graphs were produced using the ggplot2 software package [67] in R version 4.2.1 (R Core Team 2022, Vienna, Austria) [66] and prism 9.5.0 software (GraphPad, San Diego, CA, USA) for inflammation-related data analysis using paired *t*-test.

## 5. Conclusions

This study used 7 day-old biofilms on dental implants and rough titanium discs to investigate the cleansing efficacy of the Dental WaterJet with and without additional cold plasma treatment compared to cotton gauze treatment in vitro. The combined WaterJet and CAP treatment significantly improved implant surface decontamination and showed favourable results by not eliciting notable pro-inflammatory responses in human immune cells. Albeit our current study lacks in-vivo data or more complex model systems on the combination treatment, a clinical pilot study is currently in progress (German Clinical Trials Register, DRKS00026673) to explore this combination treatment in patients.

## Data Availability

The underlying data of this publication are available from the corresponding author upon reasonable request.

## Appendix A

**Table A1:** Distribution of variables presented as mean/median (25th percentile; 75th percentile) [min-max] and pairwise comparisons between groups of bacterial agar growth analysis. Groups were compared for differences using Mann-Whitney-U Test: ^a^ statistically significant difference to the negative control; ^b^ statistically significant difference to the positive control; ^c^ statistically significant difference between cotton gauze and cotton gauze + CAP; ^d^ statistically significant difference between WaterJet and WaterJet + CAP.

Group	Score	Distribution	Density	End Position
negative control (untreated biofilm)	10/10 (10; 10) ^b^	4/4 (4; 4) ^b^	4/4 (4; 4) ^b^	2/2 (2; 2) ^b^
	[10–10]	[4–4]	[4–4]	[2–2]
cotton gauze	9.2/9 (9; 10) ^a,b,c^	4/4 (4; 4) ^b,c^	3.2/3 (3; 4) ^a,b,c^	2/2 (2; 2) ^b,c^
	[7.5–10]	[4–4]	[2–4]	[1.5–2]
cotton gauze + CAP	4.7/4.3 (1; 7.5) ^a,b,c^	2.5/2.3 (0.5; 4) ^a,b,c^	1.2/1 (0.5; 2) ^a,b,c^	1/1 (0; 2) ^a,b,c^
	[0–10]	[0–4]	[0–4]	[0–2]
WaterJet	4.0/3.8 (3.5; 4.8) ^a,b,d^	1.5/1.3 (1; 2) ^a,b,d^	0.7/0.8 (0.5; 0.8) ^a,b,d^	2/2 (2; 2) ^b,d^
	[2.5–5]	[0.5–2.3]	[0.5–1]	[1–2]
WaterJet + CAP	0.5/0 (0; 0) ^a,d^	0.1/0 (0; 0) ^a,d^	0.1/0 (0; 0) ^a,d^	0.3/0 (0; 0) ^a,d^
	[0–3]	[0–0.5]	[0–0.5]	[0–2]
positive control (sterile, untreated)	0/0 (0; 0) ^a^	0/0 (0; 0) ^a^	0/0 (0; 0) ^a^	0/0 (0; 0) ^a^
	[0–0]	[0–0]	[0–0]	[0–0]

**Table A2:** Pairwise comparisons between groups (using lincom) after performing the linear regression models for associations of treatment group on all four outcomes of the bacterial agar growth analysis statistics. Beta coefficients and 95% confidence intervals are reported. Bold numbers indicate statistically significant effects (*p*-value < 0.05).

Comparison	Score	Distribution	Density	End Position
negative control vs. cotton gauze	**−0.81** **(−1.34; −0.29)**	−0.01 (−0.19; 0.17)	**−0.76** **(−1.08; −0.43)**	−0.04 (−0.17; 0.09)
negative control vs. cotton gauze + CAP	**−5.31** **(−7.04; −3.59)**	**−1.58** **(−2.43; −0.73)**	**−2.76** **(−3.35; −2.17)**	**−0.97** **(−1.41; −0.54)**
negative control vs. WaterJet	**−6.00** **(−6.60; −5.39)**	**−2.56** **(−2.95; −2.18)**	**−3.33** **(−3.47; −3.18)**	−0.11 (−0.31; 0.10)
negative control vs. WaterJet + CAP	**−9.55** **(−10.22; −8.88)**	**−3.95** **(−4.16; −3.73)**	**−3.93** **(−4.10; −3.75)**	**−1.67** **(−2.06; −1.29)**
cotton gauze vs. cotton gauze + CAP	**−4.50** **(−6.24; −2.76)**	**−1.57** **(−2.40; −0.73)**	**−2.00** **(−2.65; −1.35)**	**−0.93** **(−1.37; −0.50)**
cotton gauze vs. WaterJet	**−5.18** **(−5.90; −4.47)**	**−2.55** **(−2.93; −2.17)**	**−2.57** **(−2.90; −2.23)**	−0.07 (−0.28; 0.15)
cotton gauze vs. WaterJet + CAP	**−8.73** **(−9.52; −7.95)**	**−3.93** **(−4.14; −3.73)**	**−3.17** **(−3.51; −2.82)**	**−1.63** **(−2.03; −1.23)**
cotton gauze + CAP vs. WaterJet	−0.68 (−2.45; 1.08)	**−0.98** **(−1.89; −0.08)**	−0.57 (−1.15; 0.02)	**0.86** **(0.40; 1.32)**
cotton gauze + CAP vs. WaterJet + CAP	**−4.23** **(−6.03; −2.44)**	**−2.37** **(−3.21; −1.52)**	**−1.17** **(−1.75; −0.58)**	**−0.70** **(−1.27; −0.14)**
WaterJet vs. WaterJet + CAP	**−3.55** **(−4.39; −2.71)**	**−1.38** **(−1.78; −0.99)**	**−0.60** **(−0.78; −0.42)**	**−1.57** **(−1.99; −1.14)**

**Table A3:** Median percentages of cross points with microorganisms, organic deposits, flawless surface, scratched surface, and unknown by treatment.

Treatment	n	Microorganisms	Organic Deposits	Flawless Surface	Scratched Surface	Unknown
total						
negative control	108	88.5 (66.5; 99.8) [1–100]	0 (0; 7.3) [0–78]	0 (0; 18.8) [0–100]	0 (0; 0) [0–2.5]	0 (0; 0.5) [0–19]
cotton gauze	108	3 (1; 7.8) [0–96]	3.3 (0; 25.3) [0–91]	85 (62; 94) [0–100]	0 (0; 0.5) [0–50]	0 (0; 0) [0–4]
cotton gauze + CAP	108	4.8 (1.5; 11.5) [0–100]	5.5 (0; 30.3 [0–93]	76.8 (44.8; 94.8) [0–99.5]	0 (0; 0) [0–63]	0 (0; 0) [0–5.5]
WaterJet	108	0 (0; 0) [0–3]	0 (0; 0) [0–12]	100 (94.3; 100) [67–100]	0 (0; 4) [0–33]	0 (0; 0) [0–8]
WaterJet + CAP	108	0 (0; 0) [0–0.5]	0 (0; 0) [0–3.5]	100 (92.3; 100) [67–100]	0 (0; 7.5) [0–33]	0 (0; 0) [0–4]
positive control	84	0 (0; 0) [0–2.5]	0 (0; 0) [0–13]	100 (99; 100) [76.5–100]	0 (0; 0) [0–6]	0 (0; 0) [0–15.5]
5th implant thread						
negative control	54	88.5 (69.5; 100) [8.5–100]	0 (0; 5.5) [0–42]	0.3 (0; 21.5) [0–90]	0 (0; 0) [0–2.5]	0 (0; 0.5) [0–9]
cotton gauze	54	3.3 (1; 10) [0–96]	3.8 (0; 23) [0–91]	86.8 (61; 93.5) [0–100]	0 (0; 0.5) [0–33]	0 (0; 0) [0–2]
cotton gauze + CAP	54	4.3 (1; 15) [0–100]	3 (0; 26) [0–93]	82.3 (43; 96) [0–99.5]	0 (0; 0) [0–25]	0 (0; 0) [0–2]
WaterJet	54	0 (0; 0) [0–3]	0 (0; 0) [0–2]	100 (97.5; 100) [67–100]	0 (0; 1.5) [0–33]	0 (0; 0) [0–7]
WaterJet + CAP	54	0 (0; 0) [0–0.5]	0 (0; 0) [0–2]	100 (92.5; 100) [82–100]	0 (0; 7) [0–18]	0 (0; 0) [0–2]
positive control	42	0 (0; 0) [0–2.5]	0 (0; 0) [0–13]	100 (99.5; 100) [86–100]	0 (0; 0) [0–1]	0 (0; 0) [0–1]
3rd implant thread						
negative control	54	88.5 (55; 99) [0–100]	0 (0; 11) [0–78]	0 (0; 6.5) [0–100]	0 (0; 0) [0–1]	0 (0; 1) [0–19]
cotton gauze	54	3 (1; 5.5) [0–16]	3 (0; 28) [0–85]	84.5 (63; 94) [12–98.5]	0 (0; 0.5) [0–50]	0 (0; 0) [0–4]
cotton gauze + CAP	54	5 (2; 9) [0–87.5]	10.8 (0; 38) [0–90]	73.5 (45; 94) [0–99]	0 (0; 0.5) [0–63]	0 (0; 0) [0–5.5]
WaterJet	54	0 (0; 0) [0–0.5]	0 (0; 0) [0–12]	100 (93; 100) [73–100]	0 (0; 5) [0–27]	0 (0; 0) [0–8]
WaterJet + CAP	54	0 (0; 0) [0–0.5]	0 (0; 0) [0–3.5]	100 (92; 100) [67–100]	0 (0; 8) [0–33]	0 (0; 0) [0–4]
positive control	42	0 (0; 0) [0–1.5]	0 (0; 0) [0–2.5]	100 (99; 100) [76.5–100]	0 (0; 0) [0–6]	0 (0; 0) [0–15.5]
above implant thread (thread top land)						
negative control	36	88.5 (62; 99.3) [0–100]	1.5 (0; 7.3) [0–78]	0.5 (0; 25) [0–100]	0 (0; 0) [0–1]	0 (0; 1) [0–9]
cotton gauze	36	1 (0; 2) [0–7.5]	2 (0; 6.3) [0–45]	91.3 (77; 97.3) [47–100]	0.5 (0; 7.5) [0–50]	0 (0; 0) [0–4]
cotton gauze + CAP	36	1 (0; 4) [0–14.5]	1 (0; 6.3) [0–24]	91.8 (76.8; 96) [44–99.5]	0.3 (0; 7) [0–43]	0 (0; 0.5) [0–5.5]
WaterJet	36	0 (0; 0) [0–3]	0 (0; 0.3) [0–12]	92 (85; 94.3) [67–100]	7.5 (4; 12.5) [0–33]	0 (0; 0.5) 0–8]
WaterJet + CAP	36	0 (0; 0) [0–0.5]	0 (0; 0) [0–3.5]	88.3 (85.5; 92.5) [67–99]	11.3 (6.5; 14.5) [1–33]	0 (0; 0) [0–4]
positive control	28	0 (0; 0) [0–1.5]	0.3 (0; 1.3) [0–13]	99 (95.8; 100) [76.5–100]	0 (0; 1) [0–6]	0 (0; 0.3) [0–15.5]
underside of implant thread (bottom thread flank)						
negative control	72	88.5 (70; 100) [1–100]	0 (0; 7.5) [0–58]	0 (0; 13.3) [0–99]	0 (0; 0) [0–2.5]	0 (0; 0.5) [0–19]
cotton gauze	72	5.3 (2.3; 10) [0–96]	11.8 (0; 35.8) [0–91]	82 (48; 93) [0–98.5]	0 (0; 0) [0–1.5]	0 (0; 0) [0–1.5]
cotton gauze + CAP	72	7.3 (3.5; 14.3) [0.5–100]	17.5 (0; 41.3) [0–93]	63.8 (38.8; 93.8) [0–99.5]	0 (0; 0) [0–63]	0 (0; 0) [0–2]
WaterJet	72	0 (0; 0) [0–0.5]	0 (0; 0) [0–0]	100 (100; 100) [99–100]	0 (0; 0) [0–0]	0 (0; 0) [0–1]
WaterJet + CAP	72	0 (0; 0) [0–0.5]	0 (0; 0) [0–0]	100 (100; 100) [89–100]	0 (0; 0) [0–11]	0 (0; 0) [0–1]
positive control	56	0 (0; 0) [0–2.5]	0 (0; 0) [0–0.5]	100 (100; 100) [97.5–100]	0 (0; 0) [0–1]	0 (0; 0) [0–0.5]

**Table A4:** Median percentages of cross points with microorganisms, organic deposits, flawless surface, scratched surface, and unknown by treatment for all combinations of implant thread (3rd and 5th) and side of implant thread (underside and above). Data are presented as median (25% percentile; 75% percentile) and [min–max]. CAP, cold atmospheric pressure plasma.

Treatment	n	Microorganisms	Organic Deposits	Flawless Surface	Scratched Surface	Unknown
5th implant thread/underside of implant thread (thread flank)						
negative control	36	90.5 (77; 100) [8.5–100]	0 (0; 6.3) [0–42]	0 (0; 15.5) [0–90]	0 (0; 0) [0–2.5]	0 (0; 0) [0–5]
cotton gauze	36	6.8 (2.8; 13) [0–96]	14 (0; 35.8) [0–91]	77.5 (47; 91.3) [0–98]	0 (0; 0) [0–1]	0 (0; 0) [0–1]
cotton gauze + CAP	36	9.5 (3.3; 27.3) [0.5–100]	13 (0; 33.8) [0–93]	67.5 (28.3; 90.5) [0–99.5]	0 (0; 0) [0–1]	0 (0; 0) [0–2]
WaterJet	36	0 (0; 0) [0–0]	0 (0; 0) [0–0]	100 (100; 100) [99–100]	0 (0; 0) [0–0]	0 (0; 0) [0–1]
WaterJet + CAP	36	0 (0; 0) [0–0.5]	0 (0; 0) [0–0]	100 (100; 100) [89–100]	0 (0; 0) [0–11]	0 (0; 0) [0–1]
positive control	28	0 (0; 0) [0–2.5]	0 (0; 0) [0–0.5]	100 (100; 100) [97.5–100]	0 (0; 0) [0–1]	0 (0; 0) [0–0.5]
5th implant thread/above implant thread (thread top land)						
negative control	18	84 (62.5; 99) [44.5–100]	0.5 (0; 4) [0–26.5]	9.5 (0; 27) [0–42]	0 (0; 0) [0–1]	0 (0; 1) [0–9]
cotton gauze	18	1 (0; 2) [0–7.5]	1 (0; 4.5) [0–36.5]	92.3 (91; 98.5) [61–100]	0.3 (0; 2) [0–33]	0 (0; 0) [0–2]
cotton gauze + CAP	18	1 (0; 4) [0–14.5]	0.8 (0; 3.5) [0–10.5]	95 (88; 98) [71–99.5]	0 (0; 1) [0–25]	0 (0; 0.5) [0–2]
WaterJet	18	0 (0; 0) [0–3]	0 (0; 0) [0–2]	92.8 (92; 97.5) [67–100]	5.5 (1.5; 8) [0–33]	0 (0; 1) [0–7]
WaterJet + CAP	18	0 (0; 0) [0–0.5]	0 (0; 0) [0–2]	90 (87; 94) [82–99]	9.8 (5; 13) [1–18]	0 (0; 0) [0–2]
positive control	14	0 (0; 0) [0–0.5]	0.3 (0; 1) [0–13]	99.5 (99; 100) [86–100]	0 (0; 0) [0–1]	0 (0; 0) [0–1]
3rd implant thread/underside of implant thread (bottom thread flank)						
negative control	36	88.5 (57.5; 98.8) [1–100]	0 (0; 13) [0–58]	0 (0; 6) [0–99]	0 (0; 0) [0–1]	0 (0; 1) [0–19]
cotton gauze	36	4.5 (2; 8) [0–16]	3.5 (0; 40) [0–85]	85.5 (50; 94.3) [12–98.5]	0 (0; 0) [0–1.5]	0 (0; 0) [0–1.5]
cotton gauze + CAP	36	6 (4.3; 11.3) [1.5–87.5]	30.3 (0; 46.5) [0–90]	57.5 (42; 94.5) [0–98.5]	0 (0; 0) [0–63]	0 (0; 0) [0–1.5]
WaterJet	36	0 (0; 0) [0–0.5]	0 (0; 0) [0–0]	100 (100; 100) [99–100]	0 (0; 0) [0–0]	0 (0; 0) [0–1]
WaterJet + CAP	36	0 (0; 0) [0–0]	0 (0; 0) [0–0]	100 (100; 100) [99–100]	0 (0; 0) [0–0.5]	0 (0; 0) [0–1]
positive control	28	0 (0; 0) [0–1]	0 (0; 0) [0–0.5]	100 (100; 100) [99–100]	0 (0; 0) [0–0.5]	0 (0; 0) [0–0.5]
3rd implant thread/above implant thread (thread top land)						
negative control	18	88.5 (52; 100) 0–100	4 (0; 11) [0–78]	0 (0; 6.5) [0–100]	0 (0; 0) [0–1]	0 (0; 1) [0–3]
cotton gauze	18	1 (0; 2) 0–5.5	2.8 (1; 19) [0–45]	83.5 (72; 94) [47–98.5]	1.5 (0; 13.5) [0–50]	0 (0; 0) [0–4]
cotton gauze + CAP	18	0.5 (0; 4) 0–12	3.8 (0; 10) [0–24]	85.5 (73; 94) [44–99]	0.8 (0; 11) [0–43]	0 (0; 1) [0–5.5]
WaterJet	18	0 (0; 0) [0–0]	0 (0; 1) [0–12]	89.5 (79; 93) [73–97]	9.3 (5; 15) [0–27]	0 (0; 0) [0–8]
WaterJet + CAP	18	0 (0; 0) [0–0.5]	0 (0; 0) [0–3.5]	87.8 (85; 92) [67–96]	12 (8; 15) [4–33]	0 (0; 0) [0–4]
positive control	14	0 (0; 0.5) [0–1.5]	0.5 (0; 1.5) [0–2.5]	97.3 (94.5; 99) [76.5–100]	0.8 (0; 4.5) [0–6]	0 (0; 1) [0–15.5]

**Table A5:** Statistics of SEM analysis. Effect estimates from binomial regression models with logit link using the percentage of ‘microorganisms or organic deposits’ as the dependent variable.

	B (95% CI)	OR (95% CI)	*p*-Value	*p*-Value (Global Test)
treatment (reference: negative control)	0.00 (ref.)	1.00 (ref.)	-	<0.0001
cotton gauze	−3.473 (−4.770; −2.177)	0.031 (0.008; 0.113)	0.000	
cotton gauze + CAP	−3.811 (−5.007; −2.615)	0.022 (0.007; 0.073)	0.000	
WaterJet	−6.021 (−7.559; −4.483)	0.002 (0.001; 0.011)	0.000	
WaterJet+ CAP	−6.599 (−7.948; −5.249)	0.001 (0.0004; 0.005)	0.000	
implant thread (ref. 3rd implant thread)				
5th implant thread	0.141 (−0.790; 1.072)	1.151 (0.454; 2.921)	0.767	
side of implant thread (Ref. above implant thread)				
underside of implant thread	0.099 (−1.077; 1.275)	1.105 (0.341; 3.580)	0.868	
interaction Treatment X implant thread				0.4431
cotton gauze X 5th implant thread	−0.883 (−2.296; 0.530)	0.414 (0.101; 1.699)	0.221	
cotton gauze + CAP X 5th implant thread	−0.806 (−1.937; 0.326)	0.447 (0.144; 1.385)	0.163	
WaterJet X 5th implant thread	−1.172 (−2.990; 0.645)	0.310 (0.050; 1.907)	0.206	
WaterJet + CAP X 5th implant thread	−1.628 (−3.671; 0.416)	0.196 (0.025; 1.516)	0.119	
interaction treatment X side of implant thread				<0.0001
cotton gauze X underside of implant thread	0.850 (−0.515; 2.216)	2.341 (0.597; 9.172)	0.222	
cotton gauze + CAP X underside of implant thread	1.649 (0.272; 3.026)	5.203 (1.313; 20.624)	0.019	
WaterJet X underside of implant thread	−4.468 (−7.036; −1.900)	0.011 (0.001; 0.150)	0.001	
WaterJet + CAP X underside of implant thread	−12.699 (−14.284; −11.114)	3.05 × 10^−6^ (6.3 × 10^−7^; 0.00001)	0.000	
interaction implant thread X side of implant thread				
5th implant thread X underside of implant thread	0.345 (−1.215; 1.905)	1.412 (0.297; 6.717)	0.665	
interaction treatment X implant thread X side of implant thread				<0.0001
cotton gauze X 5th implant thread X underside of implant thread	0.743 (−1.057; 2.544)	2.103 (0.347; 12.734)	0.418	
cotton gauze + CAP X 5th implant thread X underside of implant thread	0.580 (−1.167; 2.328)	1.787 (0.311; 10.255)	0.515	
WaterJet X 5th implant thread X underside of implant thread	−8.122 (−11.021; −5.222)	0.0003 (0.00002; 0.005)	0.000	
WaterJet + CAP X 5th implant thread X underside of implant thread	10.645 (7.387; 13.902)	41962.5 (1615.4; 1090027)	0.000	
intercept	1.507 (0.444; 2.570)	4.513 (1.559; 13.061)	0.005	
